# The Progress of Surgery

**Published:** 1893-08

**Authors:** 


### Editorial, Etc.

THE PROGRESS OF SURGERY.—The history of surgery is certainly interesting. Many strange and amusing things in the progress of medical education may be studied; the low esteem in which surgeons were held, their being permitted to perform serious operations only in the presence of a physician, their prescriptions not being valid, their not being permitted to give internal medicines, the progress of the teaching of anatomy from the custom of public demonstrations once or twice a year, women licensed to practice surgery in the fourteenth century and hindered in the nineteenth, the relation that existed between barbers and surgeons who, by the way, admitted a dentist into their company in 1551, etc., etc.

The union of the barber and the surgeon in one person, or even in the same corporation, does seem strange, truly. But it was quite natural that, when bleeding was deemed necessary for the cure of most ailments, and even for the prevention of many, and when the medical ecclesiastics were forbidden to shed blood, they should turn for assistance to their barbers whom they knew to be dexterous with sharp instruments, and with basins and towels; and of course when the barbers were thus admitted to the practice of one piece of surgery, they constantly ventured further and after a time practiced many parts of minor surgery independently of the ecclesiastics. Something of this kind was really necessary because of the small number of surgeons, not ecclesiastics; for in London the whole fellowship in 1491 consisted of only eight, and in 1513 of only twelve members. And the utility of the barber-surgeon may still be observed in several parts of Europe, especially in Russia, where the fully educated surgeons are far too few for the vast and wide-spread population.

But however useful the union of surgery and "barbery" may have been, it is unfair to suppose that English surgeons are the descendants of barbers. The real surgeons held themselves apart as a distinct body. About the year 1421 they combined with the physicians. It does not appear how long this combination of the physicians and surgeons lasted. In 1493 an agreement was made between the barbers *and* surgeons, but there was no fusion of the two callings; the surgeons were not allowed to practice shaving; the barber-surgeons were not allowed to do more than draw teeth.

At last as the surgeons become more skilled and influential, and surgery became a science as well as an art, the surgeons insisted on separation. Surgery, therefore, we may believe, has always been an occupation of men who might be deemed well educated and who held good social position. R. G.

